# Pandemic or Panzootic—A Reflection on Terminology for SARS-CoV-2 Infection

**DOI:** 10.3201/eid2812.220819

**Published:** 2022-12

**Authors:** Sara Agnelli, Ilaria Capua

**Affiliations:** One Health Center of Excellence for Research and Training, University of Florida, Gainesville, Florida, USA

**Keywords:** COVID-19, SARS-CoV-2, severe acute respiratory syndrome coronavirus 2, viruses, respiratory infections, zoonoses, pandemic, panzootic, terminology, etymology, linguistics, *Suggested citation for this article*: Agnelli S, Capua I. Pandemic or panzootic, a reflection on terminology for SARS-CoV-2 infection. Emerg Infect Dis. 2022 Dec [*date cited*]. https://doi.org/10.3201/eid2812.220819

## Abstract

As of October 2022, a total of 675 natural outbreaks of SARS-CoV-2 infection have occurred in animal species worldwide. Here, we provide a linguistic and etymologic critique of the term “pandemic” being used to describe the COVID-19 health crisis, as opposed to the term “panzootic,” and discuss policy ramifications of more inclusive terminology.

As we approach the end of the third full year of the COVID-19 pandemic, the unfolding of COVID-19 continues to reveal many unexpected surprises. The most peculiar and most relevant to this paper is that we are being overwhelmed by the number of animal species which are susceptible to infection with SARS-CoV-2. As of October 2022, there have been 675 natural outbreaks in different species (*1*). A total of 58 animal species have been infected through natural and experimental infections, and these include human beings (*Homo sapiens*). Of these 58 animal species, 38 are reported in Meekins et al. (*2*) and the other 19 species are reported by other publications (*3*–*16*). Clearly, viral circulation and subsequent infection of susceptible hosts is not restricted to human beings but rather to a vast variety of animals (*1*–*13*; B. Pickering et al., unpub. data, https://doi.org/10.1101/2022.02.22.481551; S. Mahajan et al., unpub. data, https://doi.org/10.1101/2022.01.11.475327; L. Ulrich et al., unpub. data, https://doi.org/10.1101/2020.12.24.424203). 

We argue that we could be at the very beginning of a macrocycle that may become the first real-time documented case of a true panzootic—that is, an infection that occurs in a vast number of animal species, which includes *Homo sapiens*. The situation is worrisome and monitored by international organizations such as the World Organization for Animal Health and a recent effort, SARS-ANI, is collecting relevant data on animal infections in a global open-access database (*17*).

COVID-19 is currently considered a pandemic. The English term “pandemic” (*18*) comes from the ancient Greek adjective *pàndemos*, which means “of” or “belonging to” the whole people, “public” (*pan,* “all,” and *demos*, “people”). With this meaning of “public,” the word *pàndemos* already appeared in the 8th century BCE, in a passage of the *Odyssey* (*19**,* p. 200–1). Later, Plato (5th century BCE) used the term “pandemic” in the *Symposium* (*20*, p. 169–71) to describe the popular or “pandemic” love in contrast to heavenly love. Thus, the word “pandemic” with no link to any condition concerning health has been around for more than 2,800 years.

In the 2nd century CE, the Greek physician and philosopher Galen was the first to use the word pandemic in a medical treatise, although not as a medical term. In *De praesagitione ex pulsibus* 17(1).2, he refers to “the pandemic nature of famine” (*21*). Within the ancient Greek medical literature, the term “pandemic” and other terms linked to it (pandemía, pandemikós, etc.) were not associated with any specific medical condition but were used to generally describe an occurrence of variable origin and nature that was affecting “all the people.”

At its first appearance in print in England in 1666 (*22*), the word “pandemic” was essentially used as a synonym for “endemic,” which still means “a disease commonly occurring in a region or country.” “Epidemic” became the most commonly used term for large-scale infectious disease outbreaks during the 19th century until the first documented international outbreak of influenza in 1889–1891 (*23*). In a world that had known endemic and epidemic diseases, the concept of a pandemic, i.e., of a disease which could affect all the population of the world, came into shape as the 1889 influenza pandemic appeared and spread worldwide. This first occurrence was followed by the 1917–1919 Spanish influenza outbreak, which solidified the concept of pandemic, which then became accepted by the public (*24*).

In recent times, the definitions of the term “pandemic” include concepts such as “extensively epidemic” (*25*), “an epidemic occurring worldwide or over a very wide area, crossing international boundaries, and usually affecting a large number of people” (*26**,**27*), and “distributed or occurring widely throughout a region, country, continent, or globally” (*28**,**29*), among others (*24*). Although there seems to be little disagreement that a pandemic is a large epidemic, the question arises whether pandemics do include infections of other animals as well. This is not merely a linguistic matter; it has significant emergency response and policy ramifications. Policy updates would include extensive surveillance events in animals from the very start of the event to understand transmission dynamics in potential animal reservoirs.

The concept behind the term “pandemic” has evolved over the centuries to describe philosophical, social, and medical issues which had 2 common characteristics: they affected human beings and were widespread phenomena. We can say that this is certainly true also for SARS-CoV-2 infection, but “pandemic” is perhaps insufficient to encompass and define the magnitude of what we are observing with multispecies infections caused by this virus (*2*–*17*).

We believe there is another term which would perhaps be more suitable to define the extent of what we are experiencing. The term “panzootic” which literally means “all” and “animals” has been only used rarely to describe extensive multispecies infections by a single pathogen (*30**,**31*). In addition, whether *Homo sapiens* is included or not in the “-zootic” part of the word remains to be established. To look at the history of a concept through the lexicon, and particularly to the ancient Greek word ζῷον (zoon), from which the combining form -zoon comes, see Clackson (*32*). From a biological point of view, we are, of course, animals; this fact is reflected by the great number of zoonotic diseases that we are susceptible to as human beings. Some of these zoonotic events, such as HIV and swine influenza, have become pandemics.

The term “panzootic” entered veterinary and medical terminology approximately in the 19th century referring to a widespread outbreak of a disease affecting several kinds of animals. For instance, in the National Medical Dictionary (*33*), we find the entry “panzoötic,” from the neo-Latin noun panzoötia, defined as an epizootic affecting many kinds of animals. In most Romance and Germanic languages, the spelling panzoötic with umlaut changed to panzootic at the beginning of the 20th century. In the New Sydenham Lexicon (*34*), the term “panzoötic” is referring to, or the same as, panzoötia (πᾶς [pan], all; ζῶον [zoon], an animal): a disease affecting a large number of animals inhabiting extensive areas of a country.

Throughout the 19th and the beginning of the 20th century, the word panzootic had a similar meaning in its different forms within the Romance (i.e., Spanish, Portuguese, Italian, and French) and Germanic (German, English, Dutch and Swedish) languages: that is, the term used to describe a disease affecting a high number of animals in large geographic areas. This definition could be linked to the massive outbreaks of deadly diseases among animals, which were caused by highly transmissible pathogens. For example, Rinderpest affected many species in many countries and caused famine and devastation in many of them (*35*).

Of particular interest for us is a study published in the Anales de la Academia de Ciencias Médicas, Físicas y Naturales de la Habana (*36*), in which tuberculosis, a significant disease for both humans and animals, is defined as a “panzootia universal.” So, this case may be the first time panzootic was used to describe a disease which infected multiple animal species, including humans. The term panzootic was not particularly successful in gaining consensus and was virtually abandoned until a few decades ago. Since the 1980s, it has been used to describe Newcastle disease, a deadly disease of multiple species of birds which occasionally spills over to mammals, including humans, with minor consequences. Terminology is very important when defining a rare event; given the current and ever-growing evidence that SARS-CoV-2 originates from the animal reservoir and has the potential of infecting multiple mammalian species including *Homo sapiens*, it would seem reasonable to consider defining this event a potential panzootic.

We identified several reasons why defining SARS-CoV-2 as a potential panzootic rather than as a pandemic from the early phases of spread could have made a substantial difference. First, surveillance in animal populations could have started earlier, and thus active surveillance in animals would have unveiled positivity at earlier stages. Early identification of animal outbreaks could have allowed implementation of some targeted intervention strategies in advance, including developing vaccines for susceptible farm or pet animals, and prevention efforts to avoid widespread infection in wild animals. As an example, as of May 2022, SARS-CoV-2 has infected multiple white deer herds in 24 states of the United States. Infection in deer (*37*,*38*) was reported more than a year after the World Health Organization declared a Public Health Emergency of International Concern on January 31, 2020. Joint research efforts between human and veterinary virologists would empower research addressing zoonoses in a coordinated manner to prevent uncontrolled spread in large animal populations that could eventually become permanent reservoirs of SARS-CoV-2 (L.C. Caserta et al., unpub. data, https://doi.org/10.1101/2022.09.02.506368). An additional reason that becomes more topical every day is that both scientists and the public need to keep in mind that this virus is capable of spreading to wild, domestic, and pet animal species, which can in turn become reservoirs of infection for other species, including humans.

Using the most appropriate word to describe an event with unknown characteristics is often more difficult than expected. In the case of SARS-CoV-2 we believe the word panzootic is a much better fit than the word pandemic for all the reasons we mention, but especially because this usage would frame a unique event in history. We will never know if this is truly a unique event or if centuries ago other pathogens have had similar multispecies transmission cycles. Now we have the tools to assess widespread transmission in humans and animals worldwide, sometimes in real time.

This catastrophic event with unique characteristics could mark a paradigm shift in the scientific terminology, which could be used to define future events properly. Not only recent infections like SARS-CoV-2 but also other infections such as highly pathogenic avian influenza, which is now infecting wild mammals such as foxes and bears, should be deemed as potentially panzootic pathogens. This paradigm shift would also be instrumental to delivering to the public concepts that must be understood and applied in everyday life for managing and preventing further multispecies spillovers and reverse-spillover events, such as those reported by Munnik et al. (*39*) and Yen et al. (*40*) from certain animals back to other animals, known as *Homo sapiens*.
